# The effects of living and learning conditions on the health-related quality of life of children and adolescents during the COVID-19 lockdown in the French Grand Est region

**DOI:** 10.1186/s12889-022-12941-3

**Published:** 2022-03-16

**Authors:** Stéphanie Bourion-Bédès, Hélène Rousseau, Martine Batt, Pascale Tarquinio, Romain Lebreuilly, Christine Sorsana, Karine Legrand, Cyril Tarquinio, Cédric Baumann

**Affiliations:** 1 Centre Hospitalier de Versailles, Service Universitaire de Psychiatrie de l’Enfant et de l’Adolescent, 78157 Versailles-Le Chesnay, France; 2 UR 4360 APEMAC (Health Adjustment, Measurement and Assessment, Interdisciplinary Approaches), University of Lorraine, Nancy, France; 3 Methodology, Data Management and Statistics Unit, University Hospital of Nancy, 54000 Nancy, France; 4 InterPsy, GRC Team, University of Lorraine, 54000 Nancy, France; 5 Pierre Janet Center, 57000 Metz, France; 6 Clinical Investigation Center, INSERM, University Hospital of Nancy, 54000 Nancy, France

**Keywords:** Children, Adolescents, Quality of life, COVID-19, Living conditions

## Abstract

**Introduction:**

COVID-19 lockdown measures resulted in children and adolescents staying and learning at home. This study investigated health-related quality of life (HRQoL) and its associated factors among youth during the first lockdown.

**Methods:**

A cross-sectional study was conducted among 8- to 18-year-olds from the French Grand Est region. Sociodemographic data and information on living and learning conditions were collected using an online survey. HRQoL was assessed using the KIDSCREEN-27. Multiple regression analysis was performed to explore factors related to low HRQoL in each dimension.

**Results:**

In total, 471 children from 341 households were included. Difficulties isolating at home were associated with low HRQoL in the psychological well-being (OR = 2.2, 95% CI: 1.2–4.0) and parent relations and autonomy (OR = 2.1, 95% CI: 1.2–3.8) dimensions. Conflicts with dwelling occupants were related to increased ORs in the psychological well-being (OR = 2.9, 95% CI: 1.9–4.6), parent relations and autonomy (OR = 2.2, 95% CI: 1.4–3.4) and school environment (OR = 2.4, 95% CI: 1.5–3.7) dimensions. Living in an apartment (OR = 1.8, 95% CI: 1.1–3.1), never leaving home (OR = 2.6, 95% CI: 1.2–5.9), having indoor noise at home (OR = 2.3, 95% CI: 1.2–4.6), and having a parent with high anxiety (OR = 1.8, 95% CI: 1.1–3.1) were associated with low HRQoL in the social support and peers dimension. Children working less than 1 h/day on schoolwork had an increased OR of 3.5 (95% CI: 1.4–9.0) in the school environment dimension.

**Conclusion:**

Living and learning conditions were associated with low HRQoL among children and adolescents during the COVID-19 lockdown. Prevention and intervention programs are needed to support youth by facilitating their interactions and improving their coping and to prepare for future waves.

**Supplementary Information:**

The online version contains supplementary material available at 10.1186/s12889-022-12941-3.

## Introduction

In 2020, as the WHO declared the SARS-CoV-2 outbreak a pandemic [1], stringent public health measures were implemented to curtail the spread of COVID-19. Due to the seriousness of the disease [2] and the upward trends in infection rates, the French government declared a population lockdown on March 12^th^ that would be imposed as of March 16^th^ to interrupt important chains of transmission [3]. National lockdown measures included closures of schools and other educational institutions; closures of restaurants, gyms, museums and other places involving potential gatherings; and travel restrictions. These measures resulting in children and adolescents staying at home to protect themselves from any person-to-person contact. A recent meta-analysis showed that the worldwide prevalence of mental disorders in children and adolescents is 13.4% under normal circumstances [4]. Previous studies in China, Europe and the USA have reported that restrictions due to COVID-19 have adversely affected children’s and adolescents’ mental health [5–7], indicating a significant increase in mental health problems during the pandemic compared to those reported in the abovementioned meta-analysis [4].

Compared with that in adults, the measurement of health-related quality of life (HRQoL) in children and adolescents is still a relatively new field of research [8], and there has been a growing interest in the quality of life of children and adolescents during the COVID-19 pandemic. HRQoL is expressed as the patient’s subjective perception of his/her satisfaction with his/her own health, and subjective perceptions are directly related to the psychosocial state of the individual [9]. There is a general consensus that quality of life should be viewed as a multidimensional construct comprising at least physical, emotional, and social components of well-being and function [10]. According to a previous systematic review, the COVID-19 pandemic had a negative impact on the HRQoL of children and adolescents [11]. Children and adolescents who have been under lockdown have been exposed to sedentary life inside their homes might have modified their health-related behaviors [12], causing an important impact on their physical health and cardiorespiratory fitness [13]. Psychological distress may influence HRQoL, especially as circumstances with social restrictions and limited social interactions can be incongruent with children’s and adolescents’ developmental tasks [14]. Additionally, during home confinement, the limited living space can affect mental health [15]. Furthermore, due to COVID-19, children and adolescents are exposed to excessive information flow as a result of the stress and anxiety of the adults around them, which might result in children’s avoidance of sharing their own concerns [16] and, in turn, impact their HRQoL. There is a large body of literature that links parental well-being and stress to children’s outcomes [17]. It is also believed that spending too much time in the home environment increases screen-oriented activities in children [9], and the impact on their HRQoL is not yet understood sufficiently. Moreover, it has been suggested that being out of school for months risks setting back children’s learning and development [17]. By performing all learning practices at home during the lockdown, the amount of time spent learning and the availability of resources to support learning have changed. The loss of school-based instructional time may have harmed children’s and adolescents’ outcomes. Despite the increased research on children during the COVID-19 pandemic, most of the studies regarding COVID-19 and children do not address learning, which is a critical issue for their development.

Based on this recent literature on the negative impact of the pandemic on the mental health of children and because the Grand Est region was one of the three French regions that was most severely affected by the COVID-19 outbreak, the children and adolescents in this area may be at particular risk for severe mental health issues and impaired HRQoL. Thus, to understand children’s and adolescents’ needs and to develop interventions to provide suitable support in the current crisis, this study aimed to a) examine children’s and adolescents’ HRQoL during the first lockdown due to the COVID-19 pandemic and b) identify whether sociodemographic characteristics, living and learning conditions, and parental anxiety were associated with children’s impaired HRQoL.

## Methods

### Design and sample

**Fig. 1:**
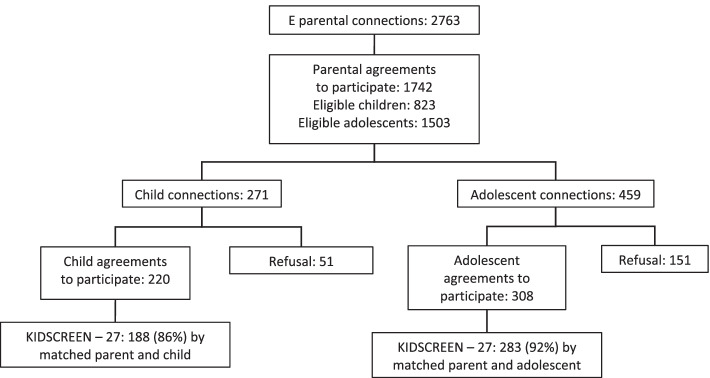
Flowchart with the selection of the population included in the study

The observational and cross-sectional study called “Feelings and Psychological Impact of the COVID-19 Epidemic among Children and Adolescents in the Grand Est Area (PIMS2-CoV19)” study was conducted from May 26th to July 6th, 2020, using an online survey while the country was under a partial lockdown. The Grand Est region incurred a high incidence of COVID-19, with 19.6 cases per 100,000 inhabitants during the survey period, being one of the three most French affected regions by the coronavirus. From a complete list of middle and high schools in the Grand Est region, schools were randomly selected using proportionate stratification for the baseline school identification and recruitment, and then the survey was disseminated through school institutional mailing lists. Children and parents were instructed to access a link containing instructions and were asked to provide their informed consent. The process for recruitment of the sample can be represented as a flow chart (Fig. 1). Among the 2763 parents who accessed the online survey, 1742 provided parental agreement regarding 823 children and 1503 adolescents. Among these children and adolescents, 271 children and 459 adolescents went online, and 220 children and 308 adolescents agreed to participate. Ultimately, completed data were obtained for 188 children and 283 adolescents. The online survey took approximately 20 min to complete. All procedures have been conducted in full compliance with the principles of the Declaration of Helsinki. Approval for the study protocol was obtained from the Commissioner for Data Protection (Comité National Informatique et Liberté-registration 2220408).

## Measures

### Health-related quality of life in children and adolescents

The KIDSCREEN-27 is a tool derived from the KIDSCREEN-52 to measure the HRQoL of children and adolescents [18, 19]. It includes five dimensions: physical well-being (5 items), psychological well-being (7 items), parent relations and autonomy (7 items), social support and peers (4 items) and the school environment (4 items). Each item is rated on the following 5-point Likert scale to assess the frequency or intensity with which the child experienced the given feeling in the last week: never (1), seldom (2), sometimes (3), often (4), and always (5) and not at all (1), slightly (2), moderately (3), very (4), and extremely (5), respectively. Negatively worded items are recoded, and higher total scores indicate better HRQoL. The French version of the questionnaire has been confirmed to have good internal consistency, with Cronbach's alpha coefficients ranging from 0.8 to 0.84, and good reliability, with values ranging from 0.61 to 0.74 [20]. Normative reference values are available for 11 European countries [21].

## Sociodemographic data and other characteristics

The survey consisted of two parts to collect parent and child data. The parent questions asked about parents’ sociodemographic information; whether parents were at home during the lockdown; whether a relative or acquaintance had been infected with COVID-19; and parental anxiety, assessed by the Generalized Anxiety Disorder-7 (GAD-7) [22, 23]. There were also questions about the child in the second part. The collected demographic data included the child’s/adolescent’s age, sex, living arrangements, home location and education level. The children were also asked about their living and learning conditions. These conditions were captured by several indicators, including objective indicators of current residence—“home location”, “type of dwelling”, and “access to a private outside space”—and subjective indicators, including “time spent on schoolwork”, “difficulty isolating at home”, “tensions and conflicts at home”, and “noises in the residence”. Children were also asked how they relieved their distress and addressed their concerns regarding the negative conditions they were experiencing. All data were obtained at the time of the online survey.

## Data analysis

### Descriptive analysis

Continuous variables are described as the means ± standard deviation (SD), and categorical variables are described as percentages. Kruskal–Wallis tests were used to compare variables between groups. The results were significant when the p value was < 0.05.

### Distribution of the KIDSCREEN-27 scores

To determine the model to be used, we first checked that there were no deviations from the normality and linearity of the KIDSCREEN-27 score distribution. Because the objective of the study was to target children and adolescents with highly impaired HRQoL, we chose the 1^st^ quartile to split the sample (i.e., impaired HRQoL vs. non-impaired HRQoL) [24].

### Bivariable and multivariable analyses

Logistic regression models were used to determine which variables were associated with the probability of having an impaired HRQoL level in each dimension of the KIDSCREEN-27. The probability modeled in each of the five dimensions was a score < the 1^st^ quartile. Sociodemographic characteristics, the influence of living and learning conditions, concerns regarding the health threat due to the COVID-19 and self-reported parental anxiety were investigated. Relevant factors were associated on bivariable analyses at the 10% threshold. Multivariable logistic regression models were then performed. The level of significance was set at 0.05. The goodness of fit was assessed by the model determination coefficient (R2), and the Hosmer and Lemeshow test allowed the comparison and selection of the best multivariable models. The dataset was analyzed using SAS 9.4 (SAS Inst., Cary, NC, USA).

## Results

### Sociodemographic, learning and living characteristics

A total of 471 children from 341 distinct households participated. Table 1 shows the sociodemographic, living and learning characteristics of the children. More than half of the children were female (53.5%), and the mean age was 12.9 (SD = 3.0). Adolescents aged 12 to 18 years represented 60% of the sample. One-quarter of the sample (25.7%) reported less than 2 h/day of homework practice during the lockdown. Media entertainment (76.7%) and physical exercise (54.9%) were the most common ways of relieving distress. Conflicts with family members who resided with the children during the lockdown were present for 29.1% of the sample, and difficulties isolating at home were reported by 13.2% of the sample. Of the 341 households, less than two-thirds lived (61.4%) in rural areas, and 6.5% reported having no access to outdoor areas. Both parents lived in the same home for two-thirds (67.8%) of the households. More than one-third of the households (38.1%) stated that a relative or acquaintance had been infected with COVID-19, and 12.3% stated that someone in their home had been confirmed to be infected with COVID-19. Regarding the coronavirus outbreak, it was found that 18% of the parents were anxious, with a GAD-7 score higher than 10 [22].

**Table 1:** Sociodemographic data, living and learning conditions of the study sample (*N*=471 children)

	**Full sample**	
	N	%/Mean (SD)
**Sociodemographic and learning characteristics (*N*=471 children)**		
**Sex**		
Male	219	46.5
Female	252	53.5
**Age**	471	12.9 (3.0)
**Education level**		
Primary school	187	39.7
Middle school	171	36.3
High school	113	24.0
**Time spent on schoolwork at home (missing=5)**		
<1 hour a day	23	4.9
1-2 hours a day	97	20.8
2-4 hours a day	209	44.8
≥4 hours a day	137	29.4
**Difficulty isolating at home**		
No	409	86.8
Yes	62	13.2
**Tensions and conflicts at home**		
No	334	70.9
Yes	137	29.1
**Noises inside the residence **		
No	428	90.9
Yes	43	9.1
**Physical exercise (missing=4)**		
Not used	58	12.4
Ineffective	18	3.9
Not very effective	49	10.5
A bit effective	86	18.4
Effective	132	28.3
Very effective	124	26.6
**Parental GAD-7 score**		
score < 10	386	82.0
Score ≥ 10	85	18.0

Abbreviation: *SD* standard deviation

### HRQoL scores of the children and adolescents during the pandemic and the lockdown

**Fig. 2:**
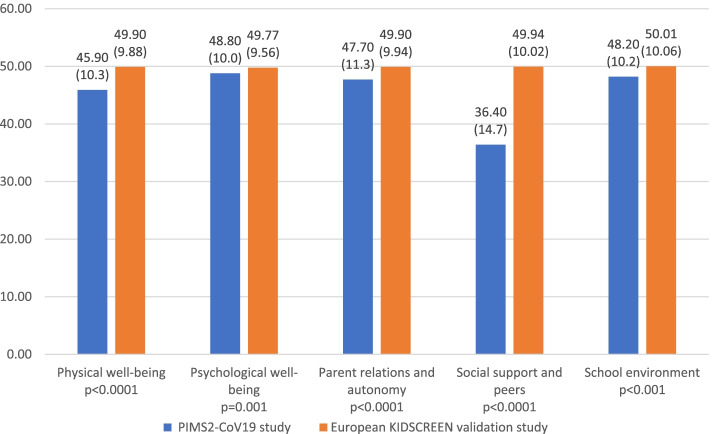
Distribution of sub-dimension average scores of KIDSCREEN-27 for children and adolescents

The average self-reported quality of life scores of the whole sample are shown in Fig. 2. For the KIDSCREEN-27 subdimensions, the highest average score was 48.8 (SD = 10.0) for psychological well-being, while the lowest average scores were 36.4 (SD = 14.7) for social support and peers and 45.9 (SD = 10.3) for physical well-being. A comparison of the mean HRQoL scores by gender and age group (8–11 years/12–18 years) is presented in Table 2. The self-reported quality of life scores for physical well-being, psychological well-being, and school environment were significantly lower in the older girl group (12–18 years) than in the other groups (physical well-being *p* < 0.0001; psychological well-being *p* < 0.0001; school *p* < 0.016). With regard to social support and peers, the scores were significantly lower for the girls in the 8–11 year group than for the other groups (*p* < 0.0001). No significant difference was found between groups in the parent relations and autonomy dimension (*p* = 0.19).

**Table 2:** Living environment characteristics (*N*=341 households)

	**Full sample**	
	N	%/Mean (SD)
**Living arrangements (missing=2)**		
Both parents	230	67.8
Single parent	19	5.6
Separated parents	90	26.6
**Home location (missing=2)**		
Urban area	131	38.6
Rural area	208	61.4
**Type of dwelling (missing=2)**		
Apartment	67	19.8
House	272	80.2
**Access to a private outside space**		
No access	22	6.5
Private balcony, courtyard or terrace	38	11.1
Private domestic garden	270	79.2
Courtyard or garden for collective use	11	3.2
**Frequency of exiting the house during the lockdown (missing=2)**		
Several times a day	36	10.6
Once a day	62	18.3
Several times a week	54	15.9
Once a week	49	14.5
Less than once a week	63	18.6
Never leaving home	75	22.1
**Someone in the household infected with COVID-19**		
No	299	87.7
Confirmed and hospitalized cases	3	0.9
Confirmed but non-hospitalized cases	7	2.1
Suspected cases	32	9.4
**Relative or acquaintance infected with COVID-19**		
No	211	61.9
Confirmed and hospitalized cases	28	8.2
Confirmed but non-hospitalized cases	70	20.5
Suspected cases	32	9.4

Abbreviation: *SD* standard deviation

### Factors associated with an impaired self-reported quality of life

Sociodemographic characteristics, the influence of living and learning conditions, concerns regarding the health threat posed by COVID-19 and self-reported anxiety in parents were included in multivariable logistic regression models. Only variables significantly associated with any HRQoL scores are presented in Table 3. The results show that in the physical well-being dimension, a single-parent family and a high level of parental anxiety were associated with more impaired HRQoL (OR = 2.1, 95% CI: 1.2–3.7; OR = 3.2, 95% CI: 1.9–5.6, respectively). However, when physical exercise was perceived as a very effective means to calm down, it served as a protective factor against impaired HRQoL (OR = 0.1, 95% CI: 0.02–0.1). Difficulties isolating at home (OR = 2.2, 95% CI: 1.2–4.0), conflicts with dwelling occupants (OR = 2.9, 95% CI: 1.9–4.6) and residence in urban areas (OR = 1.6, 95% CI: 1.1–2.5) were risk factors for impaired HRQoL in the psychological well-being dimension. Both difficulties isolating at home (OR = 2.1, 95% CI: 1.2–3.8) and conflicts with dwelling occupants (OR = 2.2, 95% CI: 1.4–3.4) were significantly associated with impaired HRQoL in the parent relations and autonomy dimension. Living in an apartment (OR = 1.8, 95% CI: 1.1–3.1), having indoor noise in one’s home (OR = 2.3, 95% CI: 1.2–4.6), never leaving the home (OR = 2.6, 95% CI: 1.2–5.9) and having a parent with high levels of anxiety (OR = 1.8, 95% CI: 1.1–3.1) were also associated with impaired HRQoL in the social support and peers dimension. Conflicts with dwelling occupants (OR = 2.4, 95% CI: 1.5–3.7) and less than 1 h/day or between 1 and 2 h/day of schoolwork were associated with a higher risk of having impaired HRQoL in the school environment dimension (OR = 3.5 95% CI: 1.4–9.0; OR = 2.0 95% CI: 1.1–3.7, respectively) Table 4.

**Table 3:** Children and Adolescents KIDSCREEN-27 scores by age group and gender group

			Age 8-11 years				Age 12-18 years				
	Total Sample		Boys		Girls		Boys		Girls		
	N	Mean (SD)	N	Mean (SD)	N	Mean (SD)	N	Mean (SD)	N	Mean (SD)	p*
Physical Well-Being	471	45.9 (10.3)	94	50.0 (9.9)	94	49.0 (9.6)	125	45.4 (10)	158	42.1 (9.9)	<0.0001
Psychological Well-Being	471	48.8 (10.0)	94	50.9 (8.5)	94	51.5 (9.9)	125	50.3 (10.1)	158	44.7 (9.5)	<0.0001
Parent &Autonomy	471	47.7 (11.3)	94	46.5 (8.8)	94	46.2 (10.4)	125	48.9 (11.7)	158	48.4 (12.6)	0.19
Social Support &Peers	471	36.4 (14.7)	94	32.2 (17)	94	30.6 (14.2)	125	37.1 (12.6)	158	41.8 (13.2)	<0.0001
School	471	48.2 (10.2)	94	49.4 (9.6)	94	50.5 (10.1)	125	46.4 (10.2)	158	47.4 (10.3)	0.015

*Abbreviation*: *SD* standard deviation

*Kruskal-Wallis test

**Table 4:** Factors associated with low HRQOL during the COVID-19 lockdown

Dimensions of KIDSCREEN-27	Influence factors	Multivariable logistic regression		
		Odds ratio	95% CI	*p* value
Physical well-being	Living arrangements single parent vs both parents	2.1	1.2-3.7	0.04
	Physical exercise - effective vs not used - very effective vs not used	0.2	0.1-0.5	<0.0001
		0.1	0.02-0.1	
	Parental GAD-7 score Score ≥ 10 vs score < 10	3.2	1.9-5.6	<0.0001
Psychological well-being	Home location urban vs rural area	1.6	1.1-2.5	0.02
	Tensions and conflicts at home Yes vs No	2.9	1.9-4.6	<0.0001
	Difficulty isolating at home Yes vs No	2.2	1.2-4.0	0.007
Parent relation and autonomy	Tensions and conflicts at home Yes vs No	2.2	1.4-3.4	0.0005
	Difficulty isolating at home Yes vs No	2.1	1.2-3.8	0.01
Social support & peers	Type of dwelling Apartment vs house	1.8	1.1-3.1	0.03
	Noises inside the residence Yes vs No	2.3	1.2-4.6	0.01
	Frequency of exiting the home during lockdown Never vs several times a day	2.6	1.2-5.9	0.0008
	Parental GAD-7 score Score ≥ 10 vs score < 10	1.8	1.1-3.1	0.03
School environment	Tensions and conflicts at home Yes vs No	2.4	1.5-3.7	<0.0001
	Time spent working at home <1 hour a day vs ≥4 hours a day 1-2 hours a day ≥4 hours a day	3.5	1.4-9.0	0.02
		2.0	1.1-3.7	

Abbreviations: *OR* odds ratio: the probability of a score in the dimension of KIDSREEN-27 < 1rst quartile; (ie low HRQOL); OR<1, decreased probability of score < 1rst quartile; OR>1, increased probability of score <1rst quartile; *CI* confidence interval

#### Discussion

The lockdown measures due to COVID-19 have involved widespread social isolation, generating massive changes in children’s and adolescents’ daily lives, including school closures, home confinement and social distancing. The results of this study provided important insights into children’s and adolescents’ HRQoL that can be used in the management of the disease. The results are in line with those of previous studies conducted in Europe [6, 25] that first showed that youth had significantly lower HRQoL during the COVID-19 pandemic. Although there were lower average HRQoL scores in the physical well-being, psychological well-being, school environment and parent relations and autonomy dimensions, the self-reported quality-of-life scores were generally good compared with the scores in the validation reference study [20]. Furthermore, the results showed that the mean scores decreased with increasing age for physical well-being, psychological well-being and school environment. In contrast, a consequent low level in the social support and peers HRQoL dimension was reported, with younger children being more negatively impacted by the pandemic than older children. First, this result is consistent with previous findings supporting that loneliness as a consequence of disease containment measures seems to be particularly problematic for young people [26]. Second, the fact that the lack of social support and peers particularly affected children may be because internet-mediated relationships are common for adolescents, for whom the peer group is particularly important for identity and support during this developmental stage [27, 28]. As a recent systematic review found a clear association between loneliness and mental health problems in children and adolescents, highlighting that loneliness was associated with future mental health problems up to 9 years later [26], health authorities and educational institutions should focus on developing interventions to maintain social support in young populations. Another interesting point is that girls were affected more in terms of psychological well-being and physical well-being than boys. On the one hand, this difference may be due to a difference in the level of physical activity between boys and girls in developing countries, as girls have a lower level of physical activity than boys [29]. On the other hand, regarding psychological well-being, the difference may be because girls are more worried about disease and may be more emotional, as previously reported in student studies [30] and have less ability to cope. Some previous findings have indicated that a sex effect emerged for physical and psychological well-being during the COVID-19 pandemic [31], whereas others did not find strong evidence [11]. Therefore, more studies should explore sex differences in the impact of the COVID-19 pandemic on HRQoL.

The original contribution of this study is its investigation of factors associated with HRQoL in children and adolescents in terms of both objective and subjective living conditions, including parental anxiety level. In line with a previous study, the home location and the type of dwelling were found to impact HRQoL during the lockdown [31] Among living conditions, difficulties isolating at home and conflicts with dwelling occupants were often associated with a higher likelihood of having impaired HRQoL in our study. As reported in previous studies [6, 32], long periods of social isolation during the pandemic are well known to be associated with avoidance behaviors, a deteriorating family climate and more escalating conflicts at home [11]. The family is the nucleus of child development; thus, the quality of family life becomes an aspect with great repercussions in the lives of children [33]. Furthermore, due to home confinement, children and adolescents could not turn to their grandparents or other family members to increase their overall resilience and cope with their stress [34]. Interestingly, a high level of parental anxiety was associated with children’s impaired HRQoL, which is in line with previous studies showing that parents’ and children’s mental health and stress are closely intertwined [6]. It is thus believed that identifying factors that lead to higher parental stress during the COVID-19 pandemic and developing interventions to decrease parents’ levels of anxiety will be beneficial to children and adolescents in improving their HRQoL. In line with previous work that showed that a decrease in learning time was observed in a portion of the students and had a negative effect on their mental health [30], this study showed that a decrease in learning time, as a direct consequence of the closure of schools, was associated with a negative effect on the children’s and adolescents’ HRQoL. The reasons for this learning decrease could be inherent to the educational system, with some teachers facing distance education for the first time and some having more difficulty than others in organizing distance learning. Another aspect is that home-based distance learning depends on the availability of electronic devices (such as personal computers, tablets and smartphones) and the availability of internet connections. Not every child has all of these components. Furthermore, children with internet access had so much spare time in the lockdown phase that they tended to spend their time on the internet, not only on school applications**.** This trend can be expected to exist among teens more than children, for whom “home-based distance learning” could be facilitated by the presence of parents, as younger children need more support in keeping in touch with the school via computer and technological devices and need more support during learning tasks [35]. Finally, the decrease in learning time might also be explained by children’s sleep behavior changes during the lockdown in comparison to their usual daily habits [36]. Given the uncertain course of the COVID-19 pandemic in the months and years to come, these results provide valuable information for tailored interventions aimed at promoting physical activity and maintaining a healthy lifestyle in the young population. Such information is important for parents, politicians, health authorities and educational institutions to take actions to reduce the impact of COVID-19 on the physical well-being of children and adolescents [37]. Schools can support children in physical activities by sending home exercise schedules for physical activities, as previously suggested [38].

This study has some limitations and strengths. First, the survey was conducted online, which may have resulted in selection bias. However, due to the pandemic, face-to-face interviews were not possible. Second, it was a cross-sectional study, and a prospective study could have better determined causation. Further prospective studies with larger sample sizes are needed to determine the factors that affect HRQoL. Third, data were collected only from the French Grand Est region, and a larger sample size with individuals from different areas of France is needed to generalize the results. Fourth, despite the large number of determinants included in the analyses, other factors, such as children’s sleep behavior and time of screen exposure, were not accounted for in our study and should be included in future studies. Last, the results were discussed in conjunction with the literature available since the current pandemic spread, but the number of studies examining the effects on young populations is still limited. However, this study provides invaluable information on children’s and adolescents’ HRQoL in an area of France particularly affected by COVID-19. Our results bring attention to the interesting finding that health initiatives for children and adolescents should include improvements in learning and living environments.

In conclusion, a low level of quality of life was found in the social support and peers subdimension in children and adolescents; thus, urgent attention to interventions aimed at facilitating interactions is necessary. It was shown that the low levels of some dimensions of quality of life were often due to living conditions such as difficulties isolating at home or conflicts with dwelling occupants. Our findings suggest that prevention and intervention programs need to be established to support children and adolescents in better coping and to prepare for future waves or comparable events.

## Supplementary Information

**Additional file 1.**

## Data Availability

Information on participants’ provision of online informed consent to participate in the study is available within the article and its supplementary material. The raw data were generated at the Methodology, Data Management and Statistics Unit and are available from the data manager (HR) on request: h.rousseau@chru-nancy.fr.
