# Supplementary material for: The effects of living and learning conditions on the health-related quality of life of children and adolescents during the COVID-19 lockdown in the French Grand Est region
Source: BMC Public Health. 2022 Mar 16;22:517. doi: 10.1186/s12889-022-12941-3 (PMC8926099; doi:10.1186/s12889-022-12941-3)
Supplement: Supplementary file 1 — Additional file 1. [file 12889_2022_12941_MOESM1_ESM.doc]

**The effects of living and learning conditions on the health-related quality of life of children and adolescents during the COVID-19 lockdown in the French Grand Est region**

**PIMS2-CoV-19 Study**

**Feelings and Psychological Impact of the COVD-19 Epidemic among Children and Adolescents in the Grand Est area, France.**

**CHILD VERSION / ADOLESCENT VERION**

1. TERMS OF INFORMATION AND COLLECTION OF CONSENT
2. Background note on the PIMS-CoV-19 Study read :

□ Yes □ No

1. You agree to participate in the Study:

□ Yes □ No
